# Heat-Denatured Lysozyme Inactivates Murine Norovirus as a Surrogate Human Norovirus

**DOI:** 10.1038/srep11819

**Published:** 2015-07-02

**Authors:** Hajime Takahashi, Moemi Nakazawa, Chihiro Ohshima, Miki Sato, Tomoki Tsuchiya, Akira Takeuchi, Masaaki Kunou, Takashi Kuda, Bon Kimura

**Affiliations:** 1 Department of Food Science and Technology, Faculty of Marine Science, Tokyo University of Marine Science and Technology, 4 -5-7, Konan, Minato-ku, Tokyo, 108-8477 Japan; 2 Kewpie Corporation, Sengawa Kewport, 2-5-7, Sengawa-cho, Chofu-shi, Tokyo, 182-0002 Japan

## Abstract

Human norovirus infects humans through the consumption of contaminated food, contact with the excrement or vomit of an infected person, and through airborne droplets that scatter the virus through the air. Being highly infectious and highly viable in the environment, inactivation of the norovirus requires a highly effective inactivating agent. In this study, we have discovered the thermal denaturing capacity of a lysozyme with known antimicrobial activity against gram-positive bacteria, as well as its inactivating effect on murine norovirus. This study is the first report on the norovirus-inactivating effects of a thermally denatured lysozyme. We observed that lysozymes heat-treated for 40 min at 100 °C caused a 4.5 log reduction in infectivity of norovirus. Transmission electron microscope analysis showed that virus particles exposed to thermally denatured lysozymes were expanded, compared to the virus before exposure. The amino acid sequence of the lysozyme was divided into three sections and the peptides of each artificially synthesised, in order to determine the region responsible for the inactivating effect. These results suggest that thermal denaturation of the lysozyme changes the protein structure, activating the region responsible for imparting an inactivating effect against the virus.

Human norovirus is a non-enveloped virus of the Caliciviridae family, which is known to cause acute gastroenteritis^1^,^2^. The virus infects humans through the consumption of raw or undercooked contaminated, food, through contact with the stool or vomit of an infected person^1^. Symptoms, such as nausea, vomiting, diarrhoea, abdominal pain, and mild fever are observed within 24–48 h after infection^1^,^3^. The virus is highly infectious, with as few as 10 to 100 individual viruses causing the onset of symptoms^4^.

Oysters and other bivalves have been widely reported^5^,^6^ as the principal carrier food of norovirus. However, the past few years have seen reports of food poisoning being caused due to the ingestion of unheated food products (ready-to-eat (RTE) products) such as salads^7^. This is believed to be due to the cultivation of fresh vegetables in virus-contaminated water^8^, or due to post-harvest handling with contaminants^5^,^7^. In recent years, most cases of viral food poisoning reported in Japan were caused by norovirus^9^. In addition, norovirus-related food poisoning cases are the highest, in terms of the number of patients, among all food poisoning cases, including bacterial food poisoning cases^9^.

This virus reportedly displays high environmental resistance^10^,^11^,^12^, and can survive for a long time on the surfaces of unclean utensils with leftover food particles^11^. Therefore, preventing the spread of norovirus infection necessitates an inactivating agent that can reliably damage or destroy norovirus particles.

Ethanol, which is currently the most-commonly used inactivating agent at food manufacturing sites, has little to no effect on norovirus^13^. Sodium hypochlorite is believed to be the most effective agent for norovirus inactivation^14^. However, the influence of organic matter (food ingredients) reduces or eliminates its effect^11^ and can only be applied to a limited number of fields^14^. This has necessitated the development of a highly effective inactivating agent to replace these.

A lysozyme is an enzyme that hydrolyses the peptidoglycan that make up the cell wall of gram-positive bacteria^15^. Lysozymes are found in human tears and breast milk, and are industrially extracted and purified from egg white^15^. They describe a broad range of applications, with examples of their use including food and pharmaceutical science^15^.

In recent years, Ibrahim *et al.* have reported on the expansion of the antibacterial spectrum of heat-treated lysozyme against even gram-negative bacteria^16^. This is thought to be due to the change in steric structure of the enzyme caused by thermal denaturation, which results in the expression of antibacterial activity even against non-gram-positive bacteria, due to the nature of the peptide itself^17^.

However, no previous reports have studied a potential interaction between norovirus and lysozyme. In addition, nothing is known about the norovirus-inactivating effects of thermally denatured lysozyme.

In this study, we discovered that thermally denatured lysozymes express an anti-norovirus effect. This study clarifies the relationship between lysozyme heating conditions and the inactivating effect against norovirus. We have also attempted to identify the mechanism of the inactivating effect of lysozyme, as well as the amino acid sequence responsible for this action. To our knowledge, this study is the first report on the inactivation of norovirus by a lysozyme.

## Methods

### Virus and viral culture

This study used murine norovirus (MNV-1), as a surrogate for human norovirus.

Murine macrophage cells (RAW 264.7) were infected with MNV-1 and cultured for three days at 37 °C in 5% CO_2_. Once sufficient cytopathic effect was observed, the cells were frozen and thawed four times and the virus particles eluted from the cells. The infectivity was measured by plaque assay, and the extracted virus solution was stored at −80 °C until further experimental use.

### Investigating the conditions of lysozyme thermal denaturation

The egg white lysozyme(Kewpie Corporation, Tokyo, Japan) was dissolved in distilled water in the concentration of 2% (w/v, pH6.9 ± 0.2) and was sterilised by filtration, using a 0.2 μm filter. These were subjected to heat treatment in an oil bath for 5, 10, 15, 30, and 40 min, at 80 °C, 90 °C, and 100 °C respectively. 500 μl of MNV-1 solution (approximately 6 log pfu/ml) was mixed with 500 μl of each of the heat-treated lysozyme solutions, in order to achieve a final concentration of 1%; the mixtures were reacted at room temperature for 1 min. Following this, the samples were collected and immediately diluted ten-fold with Dulbecco’s-Modified Eagle Medium (D-MEM), in order to stop the action of the lysozyme. A plaque assay was used to measure the infectivity.

### Investigating the concentration effect of the heat-treated lysozyme

The lysozyme solutions that were heated at100 °C for 30 min and 100 °C for 40 min were prepared to a range of final concentrations (1%, 0.5%, 0.25%, and 0.1%). 500 μl of MNV-1 solution (approximately 6 log pfu/ml) was mixed with the heat-treated lysozyme solutions of varying concentrations, and the mixtures were allowed to stand for 1 min. Following this, they were diluted ten-fold with D-MEM and the infectivity measured using a plaque assay.

### Plaque assay

The plaque assay was performed according to the method detailed by Gonzalez-Hernandez *et al.*^18^. Approximately 10^5^ cells of RAW 264.7 cells were dispensed to each well of a six-well plate (Falcon, Becton, Dickinson and Company, Franklin Lakes, NJ) and cultured for 19 h. Post the incubation time, the culture solutions were extracted from the plates, and diluted stepwise in order to produce samples. Each of the samples were inoculated in two wells at increments of 500 μL and incubated for 1 h at room temperature, with shaking. The inoculated samples were then extracted and overlaid with 2 mL of Dulbecco’s-Modified Eagle Medium (Wako pure chemical industries, Osaka, Japan) with 1.5% sea plaque agarose (Lonza Japan, Tokyo, Japan). These were cultured for 2 days at 37 °C in 5% CO_2_. They were then overlaid with 2 mL of 0.03% neutral red solution (Sigma-Aldrich Japan, Tokyo, Japan), and incubated for 1 h at 37 °C in 5% CO_2_. Finally, the stain solution was extracted, the plaque count measured, and the infectivity calculated.

### Confirmation of viral morphology by transmission electron microscopy (TEM)

The untreated virus solution, virus solution exposed to lysozyme, and virus solution exposed to thermally denatured lysozyme were all analysed by a TEM. Each virus solution was applied onto a 400-Cu grid carbon support film and treated with 2% uranyl acetate. Following this, they were observed using the JEOL JEM 1200EX (Jeol, Tokyo, Japan). Micrographs were obtained and the outer diameters of ten randomly selected virus particles from each test group were measured, in order to calculate the size of the virus particles from the magnification power of the microscope.

### Confirmation of the breaking of MNV and human norovirus by real-time PCR combined with propidium monoazide

Real-time PCR was used to measure the virus particle count, in order to confirm the killing of the virus by thermally-denatured lysozyme.

#### i) For MNV

MNV-1, which has an infectivity of approximately 6 log, was mixed with equal amounts of 2% lysozyme solution, as well as 2% lysozyme solution heat-treated for 30 min at 100 °C. The mixtures were incubated for 1 min at room temperature. Following exposure, the virus and lysozyme mixtures were diluted ten-fold with D-MEM, in order to stop the inactivating effect of the lysozyme. Samples were also prepared, where 2% heated lysozyme and MNV-1 were mixed in equal amounts and exposed for one hour.

Propidium monoazide (PMA) (Biotium, Inc., Hayward, CA) was added to the mix in order to archive a final concentration of 50 μM. These were incubated in the dark for 5 min at room temperature. These were then irradiated for 15 min with a 375 nm UV light. The RNA viral mini kit (QIAGEN K.K., Tokyo, Japan) was used to extract RNA from the samples, in accordance with the manufacturer’s protocol.

Using the extracted RNA as the template, the Prime Script RT reagent kit (Takara Bio, Otsu, Japan) was used to run a reverse transcription reaction. A primer (5′-TCC GTT CGT GTA GGT GCC TT-3′) designed for the 3′ terminus of the *VP2* gene of MNV-1, was used for the reverse transcription reaction. The reverse transcription reaction was run for 15 min at 42 °C and 5 s at 85 °C, on the Gene Amp 9700 thermal cycler (Life Technologies, Foster city, CA).

The resulting cDNA was subjected to a Real-time PCR, using the SYBR Premix Ex Taq II kit (Takara Bio). The PCR was performed with 2.0 μL of cDNA, 10 μL of 2 × SYBR Premix Ex Taq II, 0.8 μL of 10 μM- forward Primer(5′-GAT AGT TGG TGA CCA GTT TGG-3′), 0.8 μL of 10 μM- reverse primer(5′-GGT CTC TGA GCA TGT CCA G-3′), 0.4 μL of ROX reference dye, and 6 μL of double distilled H_2_O. The PCR was performed using the ABI PRISM 7900HT (Life Technologies). The following parameter was used: 10 s of 95 °C, 40 cycles of 5 s at 95 °C and 30 s at 60 °C. The threshold cycle number was used to quantify the number of MNV particles compared to the standard curve.

#### ii) For human norovirus

Stool specimen was collected from a patient who suffered from diarrhea caused by norovirus. The norovirus GII was detected in the stool sample using the qPCR Norovirus (GI/GII) Typing Kit (Takara Bio) according to the manufacturer′s protocol. A 1% (wt/vol) stool suspension was prepared with PBS(-) and clarified by centrifugation at 8,000 × *g* for 5 min. The suspension was used in subsequent experiments.

The viral sample was exposed to the heat-treated lysozyme solution for 1 min or 1 h. Following exposure, PMA treatment was performed as previously described. The sample RNA was extracted using the RNA viral mini kit (Qiagen K.K.) in accordance with the manufacturer’s protocol.

Using the extracted RNA as the template, the PrimeScript RT reagent kit was used to run a reverse-transcription reaction, using an oligo-dT primer. The reverse transcription reaction was run for 15 min at 37 °C and 5 s at 85 °C.

The resulting cDNA was subjected to real-time PCR, using the qPCR Norovirus (GI/GII) Typing Kit (Takara Bio) according to the manufacturer’s protocol. The genomic copy number for human norovirus was estimated by comparison to a standard curve for the GII-positive control DNA supplied with the kit.

### Determining the domain expressing the MNV-inactivating effect

The inactivation of MNV-1 or absence thereof, was confirmed using α-lactalbumin, which displays an amino acid sequence with high similarity to that of lysozyme. The α-lactalbumin, prepared to 1%, was heated for 40 min at 100 °C in an oil bath.

The heat-treated α-lactalbumin solution and MNV-1 were mixed at a ratio of 9:1, and incubated for 1 min at room temperature. The samples were recovered and immediately diluted ten-fold, in order to stop the action of the α-lactalbumin. Following this, a plaque assay was used to measure the infectivity.

The amino acid sequence constituting the lysozyme was divided broadly into three regions according to the motif of secondary structure, and the expression of MNV-inactivating peptide(s) was investigated for each region. The amino acid sequence of egg white lysozyme (accession no. AAL69327.1) was downloaded from the National Center for Biotechnology Information database and divided into three regions: Lz-P1 (23–57 residues), Lz-P2 (58–81 residues), and Lz-P3 (98–132 residues). All peptides were synthesised by Eurofins Genomics K.K. (Tokyo, Japan). The artificially synthesised peptides were attenuated to express the same concentration by molar ratio, and then introduced to the virus. Following this, the inactivation effect was measured using the same procedure as in the previous tests.

### Statistical processing

All experiments were performed in triplicate, and results are shown as mean ± standard error. Significant differences were tested by Duncan’s method using MS Excel.

## Results

### Relationship between the thermal denaturation temperature for lysozyme solution and the MNV inactivation effect

**Figure 1:**
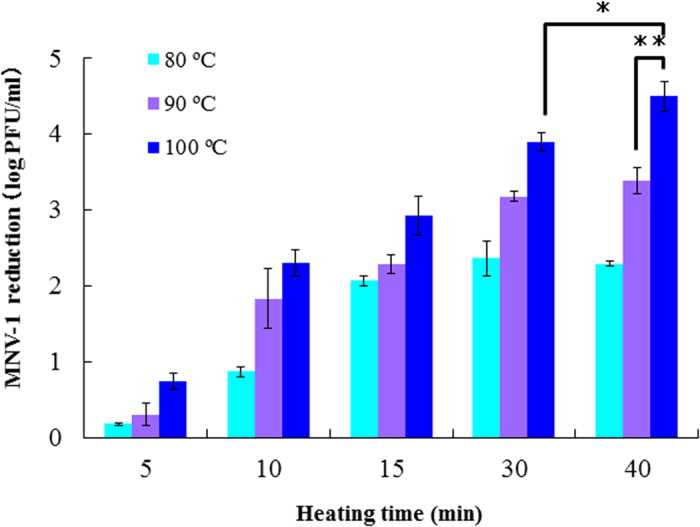
Relationship between the thermal denaturation temperature for lysozyme solution and the MNV-1 inactivation effect. Significant differences were observed between 100 °C for 40 min and 90 °C for 40 min(p < 0.01) and between 100 °C for 40 min and 100 °C for 30 min(p < 0.05).

We first studied the relationship between the thermal denaturation temperature for the lysozyme and the inactivating effect against MNV (Fig. 1). The lysozyme solution was observed to inactivate MNV by 2 log or more when heated for 15 min or longer at 80 °C and 90 °C. Lysozyme solutions denatured at 100 °C for 10 min or more were observed to inactivate at least 2 log of MNV. In lysozymes heated to 90 °C and higher, we observed a stronger inactivation effect with a longer heating time; MNV was reduced by 4.5 log by the lysozyme solution treated for 40 min at 100 °C.

### Relationship between the concentration of thermally denatured lysozyme and the MNV inactivation effect

**Figure 2:**
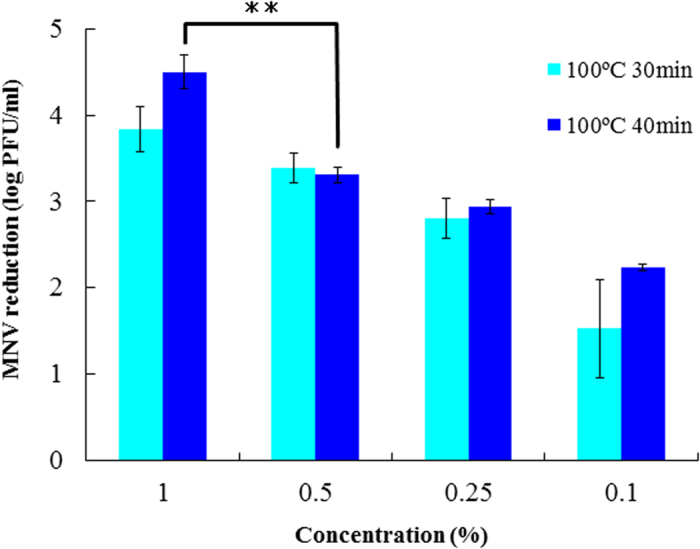
Relationship between the concentration of thermally denatured lysozyme and the MNV-1 inactivation effect. Significant difference was observed between 1% treatment and 0.5% treatment(p < 0.01).

The relationship between the concentration and the MNV inactivation effect was studied with the lysozyme solutions denatured for 30 min at 100 °C and 40 min at 100 °C (the condition that displayed the highest MNV inactivation effect in the previous test). As shown in Fig. 2, the 0.5 and 1% denatured (30 min, 100 °C) lysozyme solutions reduced MNV infectivity by 3.4 log and 3.8 log, respectively. On the other hand, the 1% denatured (40 min at 100 °C) lysozyme solution reduced the MNV infectivity by 4.5 log. Therefore, the inactivation effect against MNV was correlated to the concentration and the heating temperature of lysozyme solution used.

### Observation of the MNV exposed to thermally denatured lysozyme

**Figure 3:**
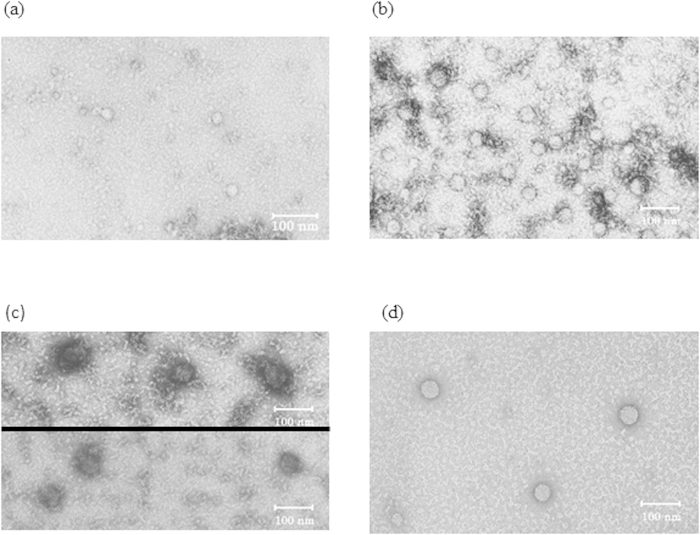
Transmission electron microscope image of (**a**) untreated murine norovirus −1(MNV-1) particles, (**b**) The MNV-1 particles exposed to the lysozyme solution for 1 min, (**c**) The MNV-1 particles exposed to the thermally denatured lysozyme for 1 min, (**d**) The MNV-1 particles exposed to the thermally denatured lysozyme for 1 hour.

The transmission electron microscopy (TEM) was used to confirm the expansion characteristics of MNV particles exposed to denatured lysozyme solution (40 min at 100 °C) (Fig. 3). In comparison to the MNV particles not exposed to the lysozyme solution, which expressed a particle size of 35.45 ± 1.70, the MNV particles exposed (1 min) to the thermally denatured lysozyme showed an average expansion of 13.64 nm (49.09 ± 2.12) (Table 1).

A greater degree of expansion was observed (51.82 ± 1.39) when the virus particles were exposed for 1 h to the thermally denatured lysozyme. However, viral particles exposed to non-denatured lysozyme solution were not significantly different (37.27 ± 1.77) from the non-exposed control particles (Table 1).

### Confirmation of the breaking of the capsid proteins of MNV and HuNV by real-time PCR combined with propidium monoazide

#### i) For MNV

**Figure 4:**
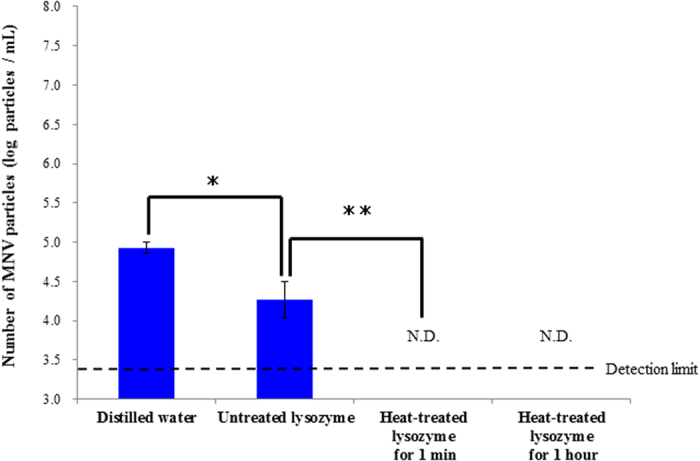
Estimation of MNV-1 particles after exposed to distilled water, untreated lysozyme for 1 min, heat denatured lysozyme for 1 min, and heat denatured lysozyme for 1 hour by real-time PCR. Significant differences were observed between distilled water and non-denatured lysozyme treatment(p < 0.05) and between non-denatured lysozyme treatment and heat denatured lysozyme treatment(p < 0.01).

Heat-treated and untreated lysozyme solutions were each mixed with MNV-1 to obtain a final concentration of 1%. Real-time PCR was used to measure the virus particle count (Fig. 4). As a result, the virus particle count in the virus samples exposed to the heated lysozyme for 1 min and 1 h were well below the detection limit of this real-time PCR assay.

The virus particle count detected after 1 min of exposure to the non-heated lysozyme was approximately 1.6 log lower than that in the control.

#### ii) For human norovirus

**Figure 5:**
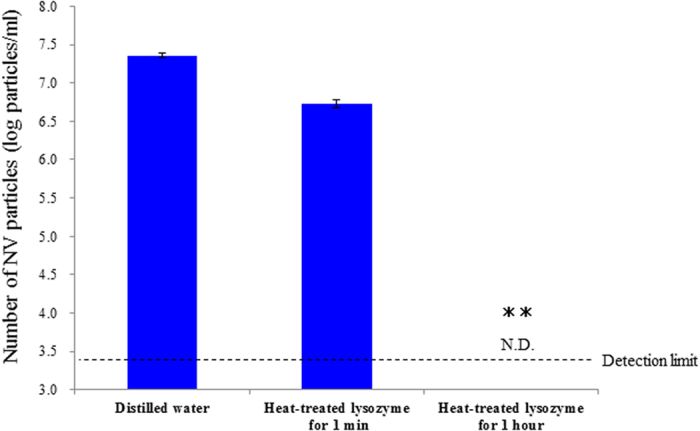
Estimation of HuNV particles after exposed to distilled water, heat denatured lysozyme for 1 min, and heat denatured lysozyme for 1 hour by real-time PCR. Significant differences were observed between distilled water and heat denatured lysozyme treatment (p < 0.01).

Heat-treated lysozyme solutions were mixed with human norovirus and the viral particles were quantified using real-time PCR (Fig. 5).

The viral particle count in the samples exposed to heat-treated lysozyme for 1 min was reduced by 0.6 log particles/ml compared to the sample exposed to distilled water. Additionally, the viral particle count in the sample exposed to heat-treated lysozyme for 1 h was well below the detection limit of this real-time PCR assay.

### Determination of the lysozyme amino acid domain expressing the MNV-inactivating effect

**Figure 6:**
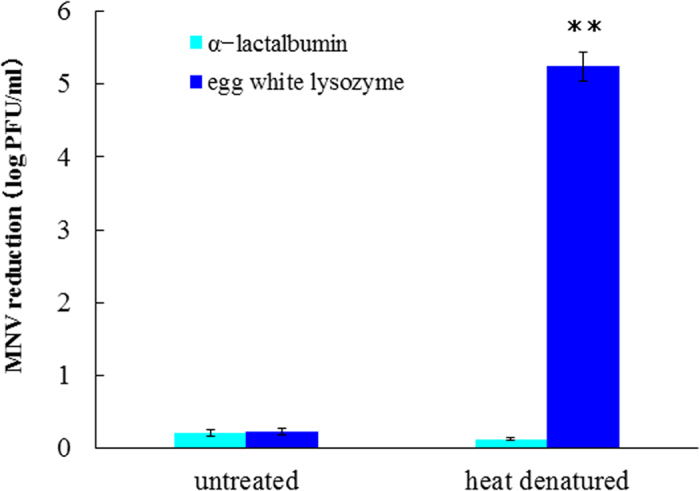
Antiviral activity of α-lactalbumin and lysozyme with heat treatment or non-heat treatment. Significant difference was observed between denatured lysozyme and lactalbmin(p < 0.01).

We first checked whether α-lactalbumin, which displays high amino acid sequence homology to the lysozyme, is capable of inactivating MNV. The MNV-inactivating ability of α-lactalbumin was approximately 0.1–0.2 log, irrespective of heat-treatment; this was significantly lower than that displayed by the thermally denatured lysozyme (Fig. 6).

**Figure 7:**
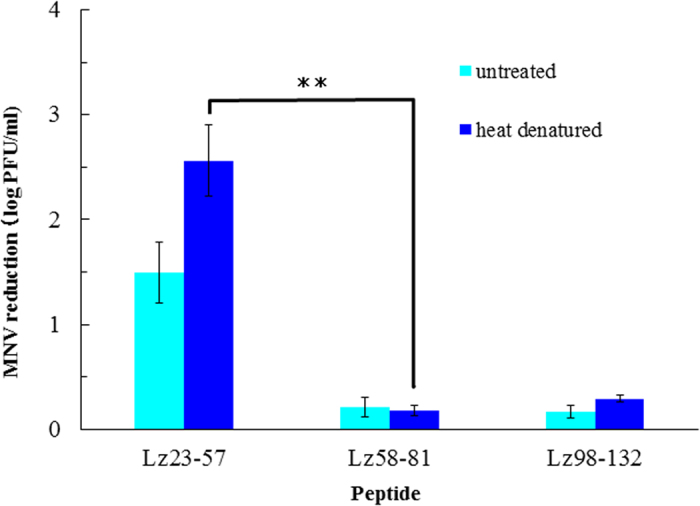
Antiviral activity of the partial amino acid sequence of Lysozyme obtained by artificial synthes. Significant difference was observed between Lz23-57 and Lz58-81(p < 0.01).

Therefore, in order to investigate the amino acid sequence of lysozyme responsible for viral inactivation, we broadly divided the amino acid sequence of the lysozyme into three sections by the motif of secondary structure of lysozyme, and studied their respective MNV-inactivating abilities (Fig. 7). Of the peptides obtained by artificial synthesis, the first set displayed a higher inactivating effect, compared to that observed in the remaining two regions (58–81 residues, 98–132 residues). This peptide region reduced the infectivity of MNV by 2.6 log. The inactivating effect of the remaining two regions was 0.2 log and 0.3 log.

## Discussion

Lysozyme is an enzyme that breaks down the cell walls of bacteria^15^, and has known antimicrobial properties, mainly against gram-positive bacteria^15^,^19^. In recent years, it has been reported that heat-treatment of lysozyme changes the steric structure of the protein, broadening its antimicrobial spectrum to include gram-negative bacteria and others^16^,^20^. It is speculated that, although heating inactivates lysozyme as an enzyme, some of its specific constituent amino acids are affected, leading to the development of antimicrobial properties^17^. In this study, we focused on this action and formulated the idea that the thermally denatured lysozyme could be used to inactivate norovirus.

Viral infectivity was successfully reduced by as much as 4.5 log when MNV, which is an alternative virus for norovirus, was exposed to the heat-treated lysozyme. A correlation was noted between the lysozyme concentration and reduction in viral infectivity, leading to the conclusion that reduced MNV infectivity was due to the lysozyme action. Because norovirus has high alcohol resistance^13^, hypochlorous acid has been generally used to inactivate this virus. However, the effect of hypochlorous acid is known to reduce due to contact with organic matter^11^. Therefore, its uses and applications in the field of food production are limited. When it is used to sterilise food products, it needs to be washed or treated prior to consumption, for safety purposes. Thermally denatured lysozyme on the other hand is safer than hypochlorous acid, because it is a protein derived from egg white. To our knowledge, this is the first report to state that thermally denatured lysozyme causes reduction of the infectivity of norovirus. Therefore, we decided to study the mechanism behind this phenomenon in further detail.

MNV exposed to heat-treated lysozyme was observed to have expanded compared to untreated MNV, by TEM analysis. Some viral particles exposed to non-denatured lysozyme were also observed to have expanded, but these were fewer in proportion. In addition, there was a significant difference between the two, when the mean particle size was taken into consideration. These observations suggest that heat-treated lysozyme effects some action on the capsid protein of the viral surface, causing expansion.

Real-time PCR investigation also showed a significant difference between virus exposed to the lysozyme and unexposed virus. We verified the destruction of the virus particles by real-time PCR, using propidium monoazide. PMA is a reagent that has been extensively used in determining bacterial cell death^21^; it flows into damaged cells, binding to the nucleic acid and inhibiting PCR. A perfect method has not been reported for the application of PMA to virus enumeration. However, Escudero-Abarca *et al.* have stated that it is effective^22^, prompting us to use it. The Ct value of the MNV-1 exposed to the heated lysozyme was delayed by six or more cycles compared to the unexposed virus, and a reduction of approximately 2 log was confirmed. There are limitations to the amount of nucleic acid that can be blocked by PMA^23^, and this test did not exactly match the effectiveness of the plaque assay. However, this technique at least confirmed the destruction of the particles of the exposed virus. The Ct value of the HuNV exposed to the heated lysozyme was also delayed, therefore, we confirmed the inactivation effect of heat-denatured lysozyme for HuNV.

Some previous studies have reported the high sequence homology between lysozyme and α-lactalbumin; the amino acid sequence homology between the two is 70%, and their secondary structures are also partially similar^24^. In order to determine whether the MNV-inactivating ability of the lysozyme is the work of the total protein structure, the constituent amino acid ratio, or a specific amino acid region, we initially compared it with α-lactalbumin. α-Lactalbumin did not display MNV-inactivating ability, regardless of heat-treatment. This led to the inference that the inactivation was the work of a specific domain, rather than the entire structure. We therefore divided the amino acid sequence of the lysozyme into three regions, focusing on the protein secondary structure. Of these, the peptide of the first region, (23rd through 57th residue), displayed a significantly higher ability to inactivate MNV than the two other peptides. In addition, no significant difference was observed between the heat-treated and non-heat-treated varieties, demonstrating significant role played by this sequence towards MNV inactivation. Therefore, it appears that heating does change the steric structure of the protein, causing the domain of this region to act on the viral capsid protein. The sequence of this region does not have a corresponding sequence in lactalbumin. This could be the reason for the inability of lactalbumin towards MNV inactivation.

This study confirms that heat-treated lysozyme displays a norovirus-inactivating effect. α-Lactalbumin, which is similar to lysozyme, displays no similar inactivating effect. In addition, only a specific region within the lysozyme expresses the murine norovirus-inactivating effect. This is a very important discovery for the future development of norovirus inactivation agents. Lysozyme extraction from egg whites is relatively inexpensive. In addition, egg white lysozymes have been regarded as a safe disinfecting agent for foodstuffs. We therefore feel that it can be extensively applied as a sanitizer, food additive, or cleaning agent for food and water.

## Additional Information

**How to cite this article**: Takahashi, H. *et al.* Heat-Denatured Lysozyme Inactivates Murine Norovirus as a Surrogate Human Norovirus. *Sci. Rep.* **5**, 11819; doi: 10.1038/srep11819 (2015).

## Figures and Tables

**Table 1:** **Particle size of MNV-1 exposed to untreated lysozyme, heat treated lysozyme for 1** **min, and heat treated lysozyme for 1 hour**.

**Exposure condition**	**Particle size (nm)**
Control	35.45 ± 1.70
Untreated lysozyme	37.27 ± 1.77
Heat treated lysozyme (1 min)	49.09 ± 2.12
Heat treated lysozyme (1 h)	51.82 ± 1.39
